# 2550. Comparing Efficacy of Serum and Epithelial Lining Fluid Concentrations for Meropenem Against Carbapenem-Resistant Enterobacterales

**DOI:** 10.1093/ofid/ofad500.2167

**Published:** 2023-11-27

**Authors:** Thomas Lavoie, Katie Daffinee, jason M Pogue, Brahim Achour, Kerry L LaPlante

**Affiliations:** Infectious Diseases Research Program, Providence Veterans Affairs Medical Center, Providence, RI; College of Pharmacy, University of Rhode Island, Kingston, RI, mattapoisett, Massachusetts; Infectious Diseases Research Program, Providence Veterans Affairs Medical Center, Providence, RI, Providence, Rhode Island; University of Michigan, College of Pharmacy, Ann Arbor, Michigan; College of Pharmacy, University of Rhode Island, Kingston, RI, Kingston, Rhode Island; 1. Infectious Diseases Research Program, Providence Veterans Affairs Medical Center, Providence, RI, United States 2. Center of Innovation in Long-Term Support Services, Providence Veterans Affairs Medical Center, Providence, RI, United States 3. College of Pharmacy, University of Rhode Island, Kingston, RI, United States 5. Warren Alpert Medical School of Brown University, Division of Infectious Diseases, Providence, RI, Kingston, Rhode Island

## Abstract

**Background:**

Carbapenem-resistant Enterobacterales are a CDC urgent threat and need effective free drug concentrations at sites of infection such as epithelial lining fluid (ELF) for pneumonia. Therefore, it’s the goal of this study to compare efficacy of meropenem serum and ELF concentrations while measuring for susceptibility changes in a five day in-vitro pharmacodynamic model.

**Methods:**

An AmpC β-lactamase producing *E. cloacae* with porin mutations and a meropenem minimum inhibitory concentration (MIC) of 2μg/mL was used to make a starting model inoculum of 6 log_10_ CFU/mL. One compartment in vitro pharmacodynamic models were run in triplicate, with inflow media set to the antibiotic half-life of 1.5 hours. Samples were taken from models for CFU/mL counts at 0, 6, 8, 24, 32, 48, 72, 96, and 120 hours with a limit of detection 2 log_10_ CFU/mL. Antibiotic target peak concentrations for serum was 26.6 μg/mL (71% T >MIC) to emulate free drug concentrations in patients at steady state receiving 2g every 8 hours. For ELF simulation, we targeted concentrations of 17.3μg/mL (58% T >MIC) to reflect ELF to unbound drug in plasma ratio of 0.65. MICs for susceptibility changes were determined via E-test, with samples from models at 24, 48, 72, 96, 120 hours being compared to 0-hour MICs. Bactericidal activity was defined as ≥3 log_10_ CFU/mL reduction in colony counts from the initial inoculum. CFU/mL counts were compared via t-test and p-values of ≤0.05 were considered significant.

Meropenem Target Concentrations

**Figure:**
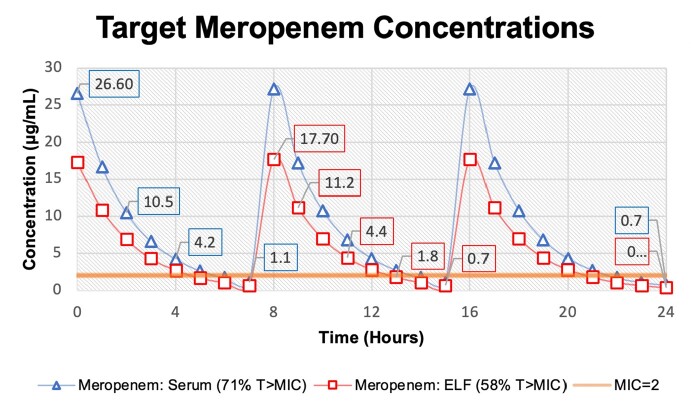

Targeted concentrations to simulate percent time of unbound meropenem in serum and ELF above the MIC

**Results:**

Serum targeted models reached the limit of detection by 24 hours with bactericidal activity through 96 hours (p< 0.05). At 120 hours, bacterial regrowth occurred in 2/3 models but was not accompanied by increased MICs (MIC=0.5μg/mL). ELF targeted models reached the limit of detection by 48 hours for 2/3 models and had significant bactericidal activity through 72 hours (p< 0.05). Regrowth occurred at 96 hours through 120 hours and was accompanied by heterogenous resistant sub-populations (MIC shifts from 2 to 4 and to >32μg/mL). The heterogenous colony formation was first seen at 24 hours in ELF models.

Meropenem Antibacterial Activity against E. cloacae

**Figure:**
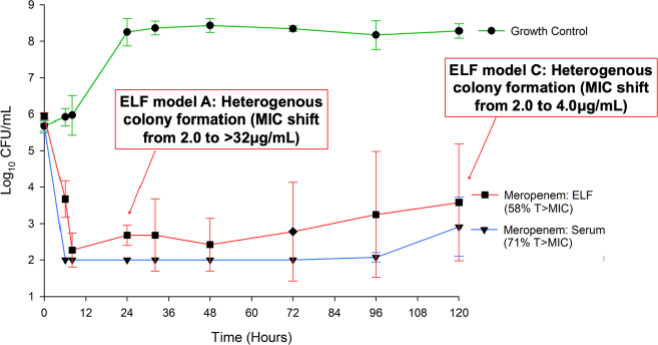

Meropenem dosing simulating serum concentrations in sick patients at steady state and in ELF

**Conclusion:**

With both meropenem targeted concentrations above recommended 40% T >MIC, there was bacterial regrowth. Target site concentrations should be considered as a contributing factor to treatment failure and resistance development.

**Disclosures:**

**jason M. Pogue, PharmD**, AbbVie: Advisor/Consultant|Entasis: Advisor/Consultant|Ferring: Advisor/Consultant|GSK: Advisor/Consultant|Merck: Advisor/Consultant|Merck: Grant/Research Support|Qpex: Advisor/Consultant|Shionogi: Advisor/Consultant **Kerry L. LaPlante, Pharm.D., FCCP, FIDSA, FIDP**, Abbive: Advisor/Consultant|Abbive: Grant/Research Support|Ferring: Advisor/Consultant|Gilead: Grant/Research Support|Melinta: Advisor/Consultant|Merck: Grant/Research Support|NIH: Grant/Research Support|Pfizer: Grant/Research Support|Seres: Advisor/Consultant|Shionogi inc.: Grant/Research Support|VA-HR&D: Grant/Research Support

